# Does scale matter? The influence of three-level spatial scales on forest bird occurrence in a tropical landscape

**DOI:** 10.1371/journal.pone.0198732

**Published:** 2018-06-18

**Authors:** Tulaci Bhakti, Fernando Goulart, Cristiano Schetini de Azevedo, Yasmine Antonini

**Affiliations:** 1 Laboratório de Biodiversidade, Departamento de Biodiversidade, Evolução e Meio Ambiente, Instituto de Ciências Exatas e Biológicas, Universidade Federal de Ouro Preto, Ouro Preto, Minas Gerais, Brasil; 2 Programa de Pós-Graduação em Análise e Modelagem de Sistemas Ambientais, Departamento de Cartografia, Instituto de Ciências Geológicas, Universidade Federal de Minas Gerais, Belo Horizonte, Brasil; 3 Laboratório de Zoologia dos Vertebrados, Departamento de Biodiversidade, Evolução e Meio Ambiente, Instituto de Ciências Exatas e Biológicas, Universidade Federal de Ouro Preto, Ouro Preto, Minas Gerais, Brasil; Sichuan University, CHINA

## Abstract

Consequences of habitat fragmentation for species occurrence are amongst the most important issues in landscape and conservation ecology. Empirical and theoretical studies have demonstrated that the total amount of habitat, patch size and connectivity have nonlinear effects on species survival on multiple spatial and temporal scales. Therefore, population models need to incorporate multiple scales, which can be extremely valuable to prioritizing conservation efforts in these changing landscapes. We tested how the amount and configuration of habitat affect understory bird species occurrence using fine to broad-scale habitat features. We used playback to sample birds in 13 Atlantic Forest fragments in Southeast Brazil. Microhabitat, local and regional landscape variables were tested against bird occurrence. Our results demonstrate that different bird species respond to different habitat scales. *Sclerurus scansor*, *Xiphorhynchus fuscus*, *Automolus leucophthalmus*, *Drymophila ochropyga*, *Mackenziaena leachii*, and *Chiroxiphia caudata* were most influenced by tree height and diameter (microhabitat characteristics), *S*. *scansor*, *F*. *serrana* and *Pyriglena leucoptera* were most influenced by forest cover and red-edge reflectance(local-scale metrics) and *S*. *scansor*, *X*. *fuscus*, *D*. *ochropyga*, *P*. *leucoptera*, *F*. *serrana* and *M*. *leachii* had area, core area and functional connectivity index (landscape features) as stronger predictors of species occurrence. Small forest fragments acted as corridors and increased overall connectivity of the entire community. The most effective means of maintaining long-term population connectivity of understory birds involves retaining both large and small areas, including forests with different micro-habitat characteristics. No management approach based on a single-scale would benefit all species. Implementing multiscale conservation strategies are necessary for maintaining long-term viability of forest birds on tropical landscapes.

## Introduction

The responses of organisms to habitat loss and fragmentation are not limited to a single spatial [1] or temporal scale [2,3], and are often associated with multiscale processes and phenomena [4]. At the local scale, forest fragmentation and degradation lead to increased mortality of large trees, particularly near edges, leading to alteration of the phytosociological structure of the forest patch [5] by forming clearings and increasing the occurrence of secondary plant species, such as lianas and vines, and this can have a negative effect for the understory birds [6,7]. On the other hand, landscape-scale characteristics are also good predictors of species occurrence, with habitat patch size and isolation (structural connectivity) being the most well-known; normally, larger areas possesses more species, and more connected fragments have more species, and both are trues especially for more habitat-sensitive birds [8]. More recently, landscape indices include functional connectivity, which associates habitat characteristics, isolation and species-specific dispersal ability to more realistically assess the response of animals to landscape features [9]. Given that connectivity is scale and habitat-dependent [9,10], it is difficult to reliably predict population response to habitat characteristics using a single scale.

The connectivity among populations of a given species is the result of the combined effects of the distribution and density of the populations, territory size, of the composition and configuration of the landscape, of the species-specific dispersal characteristics, including sex and age differences, and of the effects of different landscape features on individuals’ movement; the way such characteristics combine shapes the dispersal kernel [11]. Thus, analyses that encompass multiple spatial scales, using multiple parameters, can improve the understanding about populations’ connectivity and ecology, helping in the conservation and management of the species [9,12,13].

In order to understand the effects of habitat loss and fragmentation in disconnecting populations, it is essential to assess how the community responds to landscape changes as a function of their dispersal capacities [14]. The effects of fragmentation is more intense for species that demand large home ranges, such as some species of birds and mammals [15,16], and species with low mobility through the landscape matrix [17]. The amount of populations’ connectivity can be inferred not only by studying individual movements among fragments, but also indirectly by evaluating micro-habitat, local and landscape characteristics that allows the occurrence of that certain species in the fragments. Therefore, it is possible to estimate the effects of habitat fragmentation by evaluating the occurrence of species in the forest fragments and associating this information with multi-scale parameters, like habitat characteristics and landscape metrics.

Birds are excellent models to test the effects of fragmentation on species populations in tropical forests [18–21]. Tropical birds exhibit high species and functional richness, a wide variety of habitat use and varying degrees of sensitivity to changes in the landscape [22,23]. As a result, tropical birds have been considered good bioindicators and have been used to measure habitat quality [24–26] especially habitat specialist birds, such as forest understory insectivores [27]. Thus, studies of landscape ecology using forest birds hold the potential to contribute to the understanding and conservation of other groups of organisms.

There have been few studies on the multiscale response of birds to habitat characteristics [12,28–33] or for mammal species [11,34]. According to Boscolo and Metzger [1], landscape models that combine multiscale metrics are better predictors of bird occurrence in forest fragments than models that use a single scale. Nevertheless, these authors assessed the influence of landscape-scale metrics on different spatial scales, but did not include microhabitat. In this study, we investigated how forest bird species respond to different characteristics of three different spatial scales (microhabitat, local and landscape) in a fragmented tropical landscape using structural and functional metrics. We hypothesized that high sensitive bird species will not be using smaller, less connected forest fragments (landscape scale), will not occur in landscapes with higher urban/habitat proportion (local scale), and will not be found in areas with smaller trees and with less canopy cover (micro-habitat scale). On the contrary, low sensitive bird species will be found in more degraded areas, and medium sensitive bird species will occur in areas with intermediate characteristics.

## Methods

### Study area

**Fig 1:**
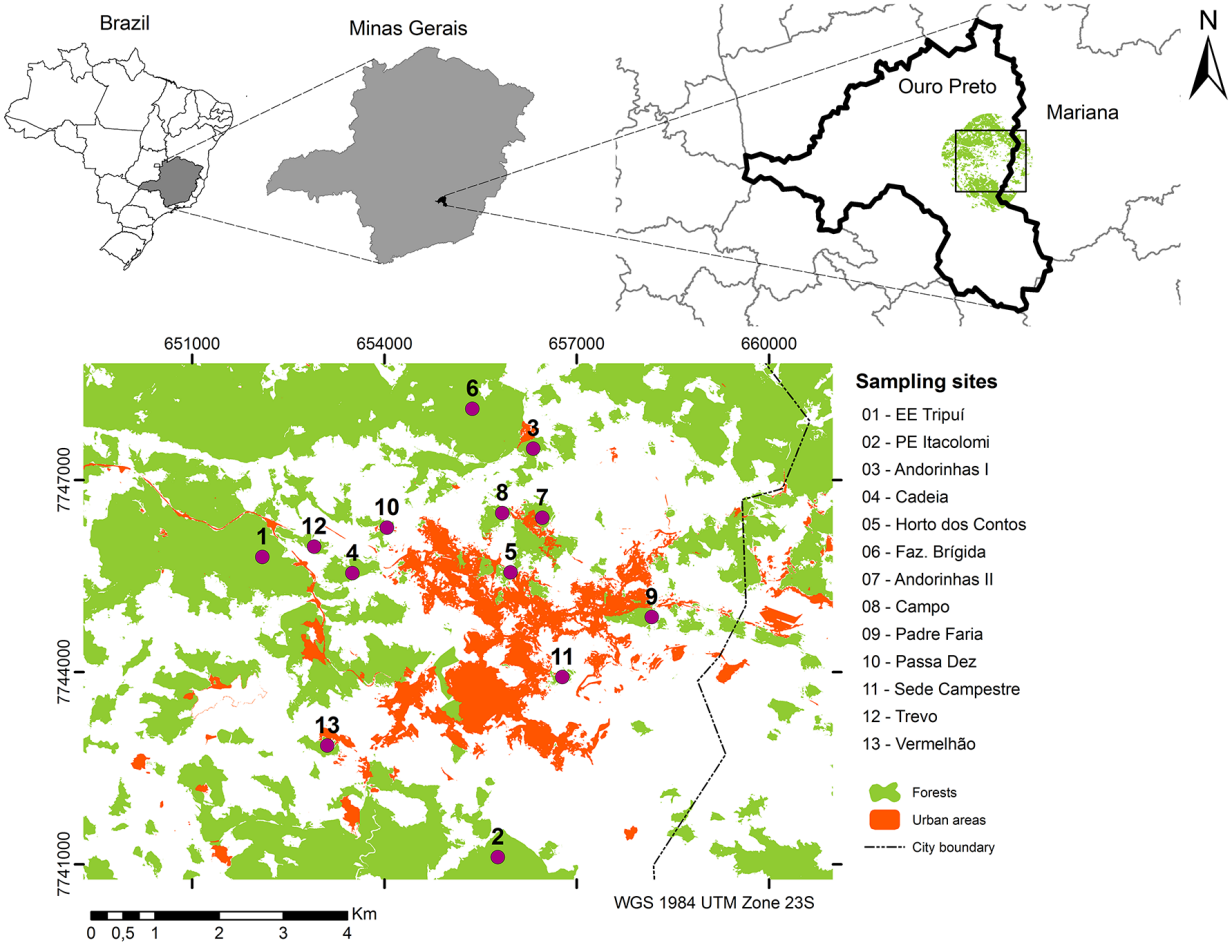
Location of the study area, localized in Ouro Preto municipality, Minas Gerais State, southeastern of Brazil—Names of the sampling areas as in S1 Table.

We surveyed understory birds within 13 public native forest patches in the municipality of Ouro Preto (Fig 1 and see S1 Table for coordinates and definitions of degree of protection for the sites), state of Minas Gerais, Southeastern Brazil. The Instituto Estadual de Florestas de Minas Gerais granted the permission to the field work (license number 064/2015) in the studies sites. The study area possesses a mosaic of natural vegetation types, including grasslands, outcrops, and forests (semidecidual forest; Atlantic Forest domain), which dominates the landscape [35], along with urban areas (Fig 1). The native forest patches varied in their sizes (4 to 6000 ha) (S1 Table). In each patch, we defined buffers of 30 m, 300 m and 5 km radius around each patch centroid to evaluate measure habitat metrics in multi-scale parameters (described below). The study was conducted from January to April 2016.

### Bird survey

Ten species of forest birds, all endemic to the Atlantic Forest domain [3,36] and previously recorded in the study region [37], were selected for the study and none are listed as threatened to extinction. These species were also selected because they show different levels of sensitivity to human disturbance [38] (Table 1). Most of the species were insectivorous, with the exception of the frugivorous *Chiroxiphia caudata* [39,40]. We also choose species for which there were enough information about dispersion in open areas habitats (flight range), as well as information on territory size [1,26,41–47].

**Table 1:** Species of endemic birds of Atlantic Forest, studied in Ouro Preto municipality, Brazil. Degree of sensitivity to anthropogenic disturbance, flight range and territory size. Breeding season: September to December^d^.

Bird Species	Common-Name	Family	Degree of Sensitivity^a^	Flight range (m)^b^	Territory size (ha)^c^
*Sclerurus scansor* (Ménétriès, 1835)	Rufous-breasted Leaftosser	Scleruridae	High	150	-
*Xiphorhynchus fuscus* (Vieillot, 1818)	Lesser Woodcreeper	Dendrocolaptidae	High	435	3,3
*Myiothlypis leucoblephara* (Vieillot, 1817)	White-browed Warbler	Parulidae	Medium	-	-
*Formicivora serrana* Hellmayr, 1929	Serra Antwren	Thamnophilidae	Medium	-	1,0
*Automolus leucophthalmus* (Wied, 1821)	White-eyed Foliage-gleaner	Furnariidae	Medium	150	5,4
*Pyriglena leucoptera* (Vieillot, 1818)	White-shouldered Fire-eye	Thamnophilidae	Medium	125	1,4
*Drymophila ochropyga* (Hellmayr, 1906)	Ochre-rumped Antbird	Thamnophilidae	Medium	-	0,83
*Mackenziaena leachii* (Such, 1825)	Large-tailed Antshrike	Thamnophilidae	Medium	-	-
*Chiroxiphia caudata* (Shaw & Nodder, 1793)	Swallow-tailed Manakin	Pipridae	Low	650	-
*Thamnophilus caerulescens* Vieillot, 1816	Variable Antshrike	Thamnophilidae	Low	80	1,3

^*a*^ [38];

^*b*^ [41,44,47];

^*c*^[45,46];

^*d*^[51,53–59]

### Unknown probably occur between Sept-Apr [50]

Bird sampling was performed using playback [48] from January to April 2016. This is a sampling technic without animal capture or manipulation and in this case, according to Brazilian Regulation for animal studies, no approval from an Institutional Animal Care is necessary.

This sampling period was chosen because it is the rainy season (summer) in the tropics, which coincides with the reproductive season of most of the bird species and also with the period of parental care [39,49,50]. Playback of species’ songs and calls were performed for 30 seconds, followed by 30-second intervals, repeated for a total duration of five minutes (adapted from [51]). All areas were sampled three times, with intervals of 30 days between visits, for a total of 78 hours of sampling. The sampling intervals of 30 days diminished the chance to count the same individuals, and the repetition of samplings expanded the possibilities to record a higher number of bird species, and this is indicated for neotropical birds [51,52]. Sampling occurred from 6:00 to 10:00 AM in each day. Sampling was not performed on rainy days, which could decrease the chances to detect the presence of the chosen bird species.

### Measured habitat metrics

**Fig 2:**
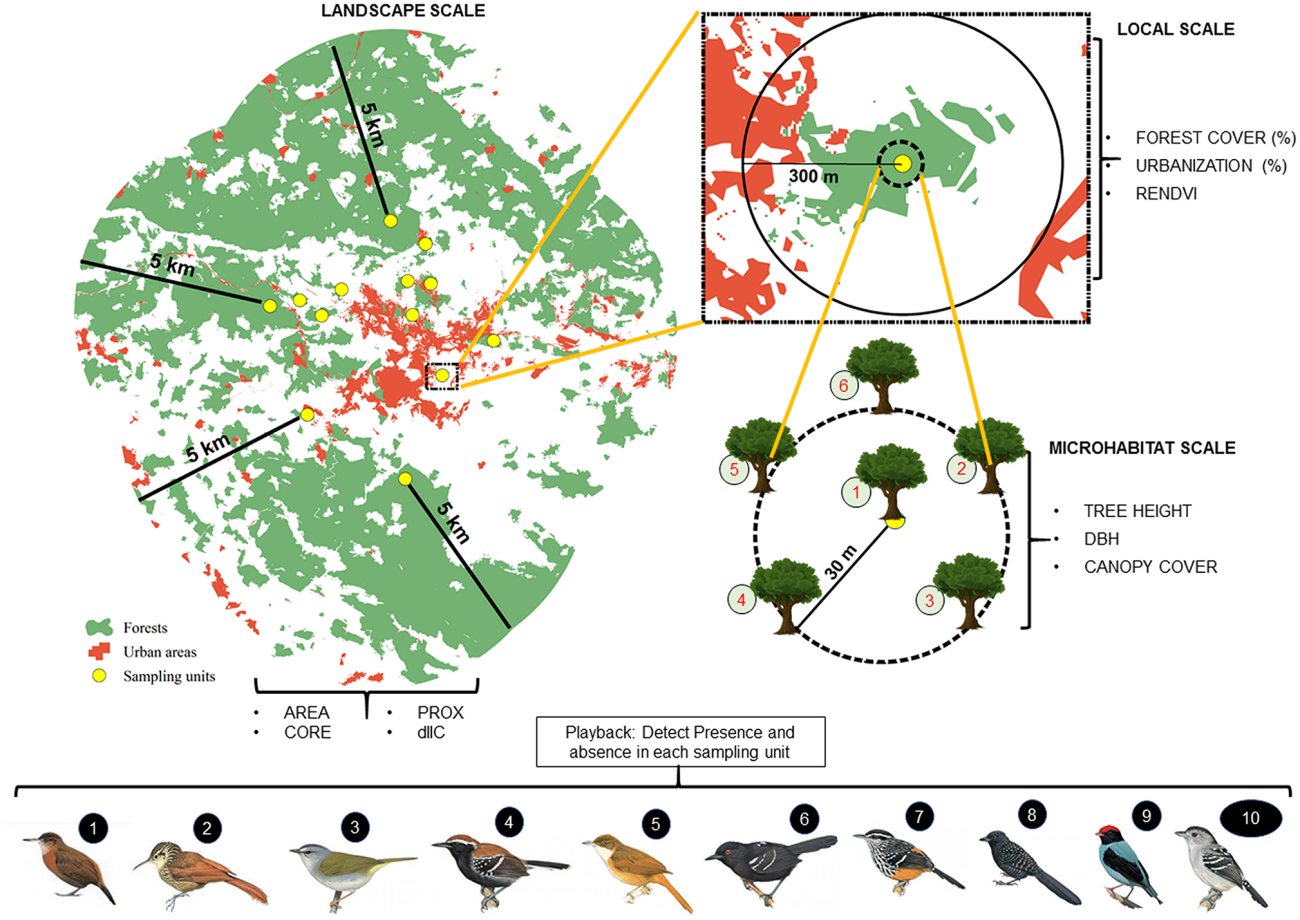
Multiscale samplings in the studied patches. In the landscape patch, 5 km buffers were used and the area, core, prox and dIIC (Integral Connectivity Index delta) characteristics were measured; in the local scale, 300 m buffers were used and the percentage of forest cover and urban area, and the ReNDVI (Red Edge Normalized Difference Vegetation Indexes) were measured; in the micro-habitat scale, 30m buffers were used and tree height, DBH (diameter of breast height) and canopy cover were measured in points at the end of the five 30 m transects positioned in North, East, Southeast, Southwest and West directions. All measures took at the three scales were used to explain the presence-absence of ten bird species (1: *Sclerurus scansor*; 2: *Xiphorhynchus fuscus*; 3: *Myiothlypis leucoblephara*; 4: *Formicivora serrana*; 5: *Automolus leucophthalmus*; 6: *Pyriglena leucoptera*; 7: *Drymophila ochropyga*; 8: *Mackenziaena leachii*; 9: *Chiroxiphia caudata*; 10: *Thamnophilus caerulescens*). Bird picture resource: http://www.hbw.com.

We quantified the percentage of natural vegetation in the study region using the ArcGIS/ArcMap version 10.4 [60], based on Rapid-eye (2011; 5-m resolution; bands 3, 4 and 5) satellite image obtained from the Ministry of Environment of Brazil [61]. We performed a supervised classification to separate forest (natural and secondary forest) and urban areas (buildings, roads and impacted areas with exposed soil) from all other land cover (outcrops, mining sites and agricultural patches). The amount of forest in the region was assumed to correspond to the availability of habitat for these forest-specialist bird species. The precision of the classification was measured using discriminant analysis [62], with individual values of 83% (forest) and 92% (urban area), and an accumulated value of 80% (sum of the individual values of all classes of land cover). For the multiscale analyses, we used three scales: microhabitat, local and landscape scale (Fig 2).

At the landscape scale, we quantified landscape structure within a radius of 5km of each forest fragment using FRAGSTATS v.4 [63] to obtain values for four variables: Area—which corresponds to the total size of each fragment; Core—nuclear area at 100 m from edge; ENN—edge-to-edge distance from nearest neighboring fragment; and PROX—proximity index, which is calculated as the areas of the fragments divided by ENN values. We also calculated the Integral Connectivity Index (IIC), which determines the individual importance percentage of each fragment in the landscape and how much each contributes to functional connectivity. This metric effectively combines the effect of patch area, isolation and species dispersal ability with delta IIC (dIIC) scores, to simulate the removal of the patch, and determine the consequence to overall connectivity [64]. To this end we used the software Conefor v.2.6 [65].

At the local scale, we quantified proportion of forest cover and urban area within a radius of 300 m of each bird survey point (Fig 2). Field trips associated with satellite images were used to verify the characteristics of each sampling areas and to quantify the categories of soil use in the studied areas. The classification of soil use was made by analyses in the ArcGis 10.4. We also produced Red Edge Normalized Difference Vegetation Indexes (ReNDVI) as a measure of habitat quality. This index varies from -1 to 1, with values close to 1 indicating areas with more forest cover [66]. ReNDVI permits the estimation of tree species density in fragmented areas [67] by enabling models that use fragmentation in forest characterization [68], and by quantifying vegetation quality based on their density and structure for associating with the landscape chlorophyll [69]. In this study, we used the mean ReNDVI values of each pixel (5m x 5m) within the buffers.

At the microhabitat scale, we sampled vegetation in five radiating transects of 30 m from the central point of sampling of birds (Fig 2). A compass and a measuring tape were used to mark the transects. Within the center point of each transect we quantified tree DBH (diameter of breast height) and height, and a detailed canopy cover metric (S2 Table). The tree variables and canopy cover represents indirect measures of resources availability for birds and of habitat quality, like foraging and nesting places [70,71]. Canopy cover is likely to be an important measure of habitat availability for forest-dependent species, and, since forest-dependent bird species are photophobic [39], evaluating habitat quality using canopy cover as a parameter is important for the studied population [10]. We thus measured canopy cover with a convex spherical densitometer (Mid-OMount and WinSCANOPY) at each corner and within each point, and then obtained a mean value for the point. Prior to analysis, we averaged the five points for each vegetation measure to characterize the local habitat of each forest fragment. These variables were measured along with the sampling of birds.

### Statistical analysis

The relation of each environmental variable to bird species occurrence in each patch was evaluated using Generalized Linear Models with binomial distribution for each scale. To test whether a species’ response to each variable at each scale varied as a function of its sensitivity to habitat fragmentation, we used the Stotz et al. (1996) classification on “sensitivity to human disturbance”. Environmental characteristics were the explanatory variables and the occurrence of bird species was the response variable. We firstly run multiple variables within one GLM model; then, we eliminate the variables with the small weight on the model, running all combinations of variables. In this procedure, we detected that the variables AREA, dIIC and CORE were highly correlated, and they were not used in subsequent analyses. The interaction between the variables Area and ENN was inserted in specific models to evaluate if the size of the area and the distance to the nearest fragment influenced the occurrence of bird species in the studied area. In total, we built 110 models. The loglikelihoods of the models were then used to calculate the Akaike information criterion corrected for small samples (AICc) [72] and Akaike weights (w), which we used in model comparisons, only those models with AICc<2.0 and p<0.005. We did not include strongly correlated variables (Spearman r > 0.50) in the same model. The analysis was performed using the package lme4 and AICcmodavg in the statistical computing software R [73].

## Results

**Fig 3:**
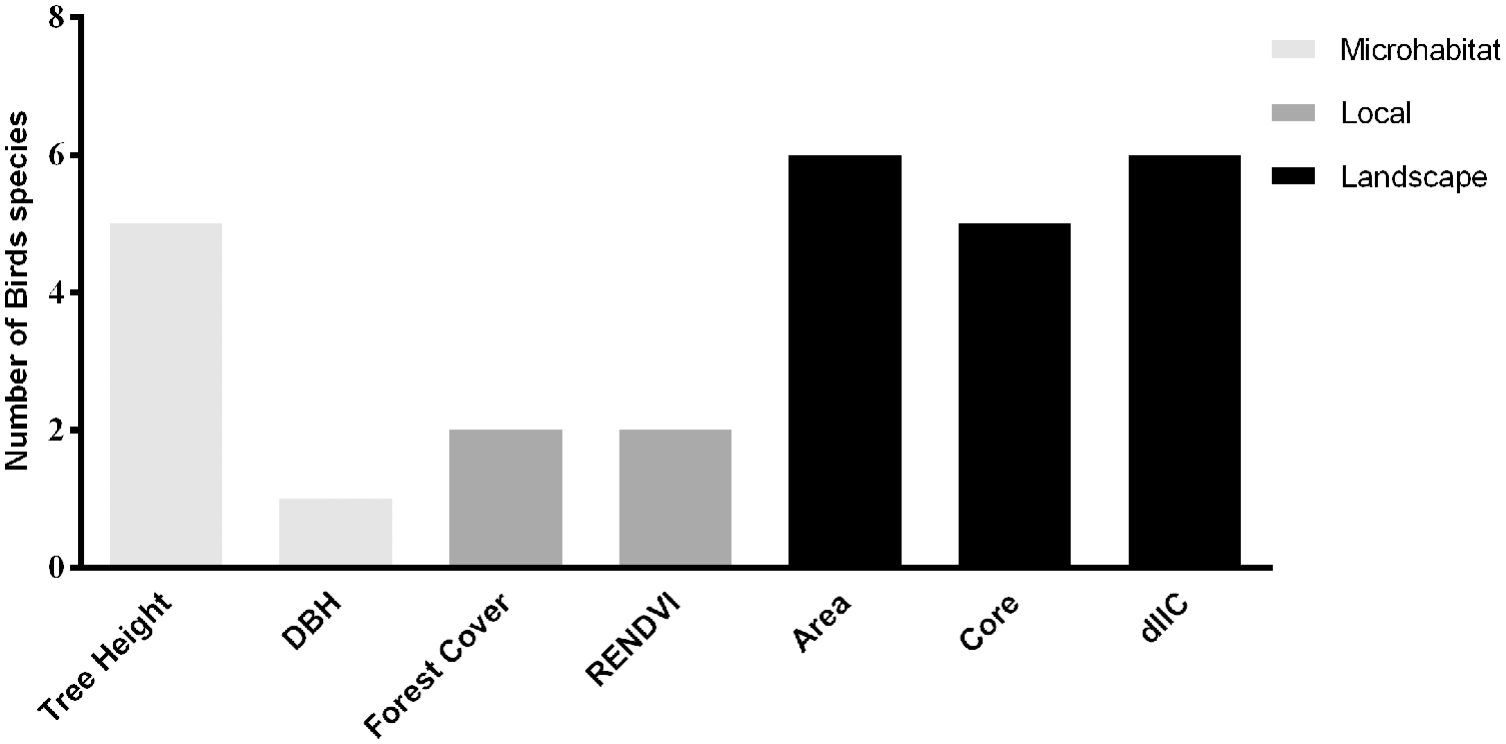
Number of bird species influenced by some of the microhabitat (light gray bars), local (dark gray bars), and landscape (black bars). For microhabitat scale: tree height: *S*. *scansor*, *X*. *fuscus*, *A*. *leucophthalmus*, *D*. *ochropyga* and *M*. *leachii*; DBH: *C*. *caudata*. For local scale: forest cover: *F*. *serrana* and *P*. *leucoptera*; ReNDVI: *S*. *scansor* and *F*. *serrana*. For landscape scale: Area: *S*. *scansor*, *X*. *fuscus*, *F*. *serrana*, *D*. *ochropyga*, *P*. *leucoptera* and *M*. *leachii*; Core and dIIC: *S*. *scansor*, *X*. *fuscus*, *F*. *serrana*, *D*. *ochropyga* and *M*. *leachii*.

The presences of six bird species were influenced by microhabitat variables, of three bird species were influenced by local scale variables and six species were influenced by landscape scale metrics (Fig 3). For all variables, except DBH, we found a positive relationship with bird species presence. At the microhabitat scale, four models were significant; six models were significant at the local scale and 16 models were significant at the landscape scale, totaling 25 significant models (Table 2).

**Table 2:** Significant General linear models (GLMs) at microhabitat, local and landscape scales. For the models, the dependent (bird species) and explanatory variables are informed. Akaike’s information criterion corrected for small samples (AICc) with delta AICc and weight wAICc (ΔAICc and w).

Scale	Model Dependent variable (bird species) ~ Independent variable	AICc	ΔAICc	w
Microhabitat	*Automolus leucophthalmus* ~ Tree Height	11.14	0	0.90
	*Chiroxiphia caudata* ~ DBH	9.31	0	0.61
	*Drymophila ochropyga* ~ Tree Height	15.65	0.4	0.23
	*Mackenziaena leachii* ~ Tree Height	13.81	0	0.335
	*Sclerurus scansor* ~ Tree Height	13.81	0	0.323
	*Xiphorhynchus fuscus* ~ Tree Height	17.12	0	0.36
Local	*Formicivora serrana* ~ Forest Cover	15.12	1.5	0.138
	*Formicivora serrana* ~ ReNDVI	13.61	0	0.295
	*Pyriglena leucoptera* ~ Forest Cover	5.20	0	0.478
	*Sclerurus scansor* ~ ReNDVI	15.40	1.6	0.146
Landscape	*Drymophila ochropyga* ~ Area	15.49	0.2	0.25
	*Drymophila ochropyga* ~ Core	15.26	0	0.28
	*Drymophila ochropyga* ~ dIIC	15.45	0.2	0.25
	*Formicivora serrana* ~ Area	15.08	1.5	0.141
	*Formicivora serrana* ~ Core	15.24	1.6	0.130
	*Formicivora serrana* ~ dIIC	14.30	0.7	0.208
	*Mackenziaena leachii* ~ Area	15.28	1.5	0.161
	*Mackenziaena leachii* ~ Core	15.21	1.4	0.166
	*Mackenziaena leachii* ~ dIIC	15.30	1.5	0.159
	*Pyriglena leucoptera* ~ Area	5.20	0	0.478
	*Sclerurus scansor* ~ Area	15.36	1.6	0.149
	*Sclerurus scansor* ~ Core	15.26	1.5	0.156
	*Sclerurus scansor* ~ dIIC	15.21	1.4	0.160
	*Xiphorhynchus fuscus* ~ Area	18.25	1.1	0.20
	*Xiphorhynchus fuscus* ~ Core	17.95	0.8	0.23
	*Xiphorhynchus fuscus* ~ dIIC	18.18	1.1	0.21

The presences of five species were positively related to tree height: *Sclerurus scansor*, *Automolus leucophthalmus*, *Drymophila ochropyga*, *Mackenziaena leachii* and *Xiphorhynchus fuscus*, while the presence of *C*. *caudata* was related to DBH values (Table 2). At the local scale, the presences of *Formicivora serrana* and *Pyriglena leucoptera* were positively related to forest cover; while *F*. *serrana* and *S*. *scansor* were positively related to ReNDVI (Table 2). The presences of six bird species were positively related to landscape scale variables (Table 2). For *D*. *ochropyga*, *F*. *serrana*, *M*. *leachii*, *S*. *scansor* and *X*. *fuscus*, Area, Core and dIIC were the best predictors of occurrence, and for *P*. *leucoptera*, AREA was the best predictors of occurrence. None of the variables were significantly associated with the occurrence of *Myiothlypis leucoblephara* or *Thamnophilus caerulescens* at all scales. The variables canopy cover (microhabitat scale); proportion of forest cover and urban area (local scale), and PROX, ENN (landscape scale) were not significantly related to any presence of the bird species.

**Fig 4:**
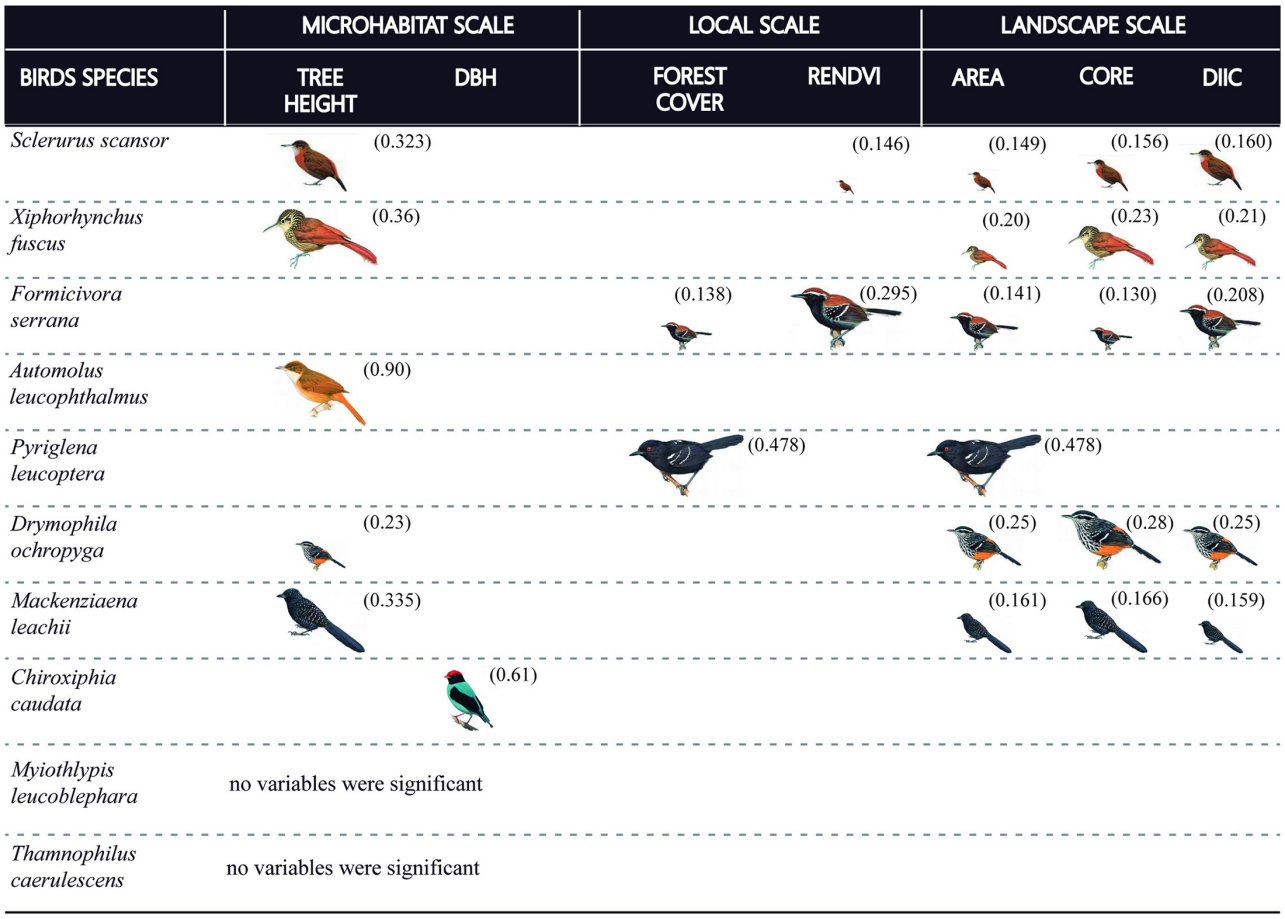
Influence of each habitat variable in the occurrence of the evaluated bird species in relation to the wAICc values. The size of each bird varies according to wAICc values. That is: the smaller the bird picture, the lower the wAICc value. wAICc values were demonstrated in the figure beside each bird representation. The absence of a wAICc values of some of the 10 bird species in this table means no significant influence of that variable on its occurrence (p>0.05). Bird picture resource: http://www.hbw.com.

The presence of *A*. *leucophthalmus* was related to fragments with taller trees (w = 0.90), and *C*. *caudata* was related to DBH (w = 0.61), these two parameters being the most important predictors to their presence (Fig 4). The presence of *D*. *ochropyga* was strongly related to the nuclear area (w = 0.28), to fragment size and connectivity (w = 0.25), and taller trees (w = 0.23) (Fig 4). The presence of *F*. *serrana* was influenced by the forest quality (w = 295), the connectivity and the patch size (w = 0.208 and w = 0.141), forest cover (w = 138) and nuclear area of the fragment (w = 13) (Fig 4).

Tree height (w = 0.335), nuclear area (w = 0.166), fragment size (w = 0.161), and connectivity degree (w = 0.159) significantly affected the presence of *M*. *leachii* (Fig 4). The occurrence of *P*. *leucoptera* was influenced by patch size (w = 0.47) and forest cover (w = 0.47) (Fig 4), while that of *S*. *scansor* was strongly related to the tree height (w = 0.32), connectivity degree (w = 0.16), nuclear area (w = 0.156) and patch size (w = 0.149) (Fig 4). For *X*. *fuscus*, its presence was affected by tree height (w = 0.36), nuclear area (w = 0.23), degree of connectivity (w = 0.21), and patch size (w = 0.20) (Fig 4).

## Discussion

The occurrence of the selected bird species was influenced by a variety of habitat characteristics, and multiscale evaluation proved to be an important tool for providing valuable information for bird conservation. Although local and landscape scale variables were important for predicting site occupancy by the selected species, their specific responses varied in relation to the variables of each scale. Thus, studies that aim to evaluate the effects of fragmentation on multiple scales are fundamental for acquiring important information and for the conservation of forest birds in the Neotropical region.

We hypothesized that high sensitive bird species would not be using smaller, less connected forest fragments (landscape scale), would not occur in more will not occur in will not occur in landscapes with more urban-habitat ratio proportion (local scale), and would not be found in areas with smaller trees, with less canopy cover (micro-habitat scale). Our results showed that these species can occur in smaller areas, but these areas need to have higher trees and needs to be more connected to other fragments. Vegetation density and the amount of forest cover were not good predictors for the most sensitive species. We also predicted that low sensitive bird species would be found in more degraded areas, and that medium sensitive bird species would occur in areas with intermediate characteristics, and our results showed to partially corroborate these ideas. Among the lowest sensitive birds, only *C*. *caudata* showed to be influenced by a micro-habitat characteristic (DBH), with this species occurring in areas with higher DBH trees. The medium sensitive birds varied in their responses to multi-scale parameters, but in general, landscape parameters, like size and connectivity, influenced these species the most.

No single scale could be used to predict the occurrence of all studied bird species. Hence, conservation strategies that rely on a single spatial scale would be unsuccessful in conserving all of the selected species. Our results indicate that in landscapes with an intermediate proportion of remaining forest, with small forest patches that provide connectivity and form corridors and/or stepping stones, could be more important than the fragment size (i.e., the local amount of habitat) in determining species presence. Martensen et al. [74] found similar results.

For instance, for some of the selected species, the presence was more influenced by microhabitat variables, than landscape characteristics. For these species, the occurrence was determined by local characteristics, such as vegetation height and DBH; forest patches with large and tall trees are normally better conserved (primary forests) than areas without trees with such characteristics [75]. These trees can provide food, shelter and nesting places for the birds, and can sustain more bird species than areas without these trees [76]. Forest birds are highly susceptible to microclimatic alterations, vegetation characteristics (like vegetation density, the existence of understory and the proportion of canopy cover) and reductions in food resources and nesting sites [18,30,77]. Thus, microhabitat characteristics may be a better feature to suggest habitat quality.

The presence of *A*. *leucophthalmus* in the studied fragments was related only to tree height. This variable, however, was also related to the presence of four other species (*M*. *leachii*, *D*. *ochropyga*, *S*. *scansor* and *X*. *fuscus*). In three of these species, tree height was of the highest weight value (w) compared to the other variables, indicating the great importance of this microhabitat variable. According to Martensen et al. [74], the amount of forest, as well as its structure, affects the occurrence of understory bird species, with greater bird richness being found in landscapes with a larger proportion of forest associated with greater canopy cover. The height of the trees could favor greater vegetation cover and a possible stratification of foliage that permit positive habitat for bird species [32], like *S*. *scansor*, which is very sensitive to luminosity and possesses photophobia, and thus inhabits darker portions of the forest [39]. Pollock et al. [78] also argued that the presence of understory vegetation is a determinant for the occurrence of forest birds. Our results corroborate those authors. The richness of understory birds was higher in patches with a higher proportion of preserved forests, even in the cases where these patches are surrounded by a less permeable matrix [79,80]. It should also be mentioned that the presence of bird species in our study sites might be favored by the proximity of conservation units that can function as a source of individuals for the patches in the landscape [81].

On a local scale, the presences of *F*. *serrana*, *P*. *leucoptera* and *S*. *scansor* were related to proportion of forest and local greenness (given by ReNDVI). Therefore, for *F*. *serrana*, the greater amount and density of forests, the higher the probability of occurrence, indicating a high dependence on forests. The occurrence of *P*. *leucoptera* was also influenced by the fragment size and the amount of forest, which had been previously reported for other regions of the Atlantic Forest [1].

Although we expected a positive relationship between the landscape scale variables Area, Core and dIIC and the presence of bird species, this occurred only for six of the studied species [*S*. *scansor*, *X*. *fuscus*, *D*. *ochropyga*, *P*. *leucoptera* (only Area), *F*. *serrana* and *M*. *leachii*]. At the landscape scale, the importance of fragment size and structural/functional connectivity varies as a function of forest amount, and there seems to be a fragmentation threshold for highly sensitive species. According to Martensen et al. [74], moderately sensitive species were particularly affected by connectivity in landscapes with more forest cover.

Therefore, larger forest fragments have larger nuclear areas and possess greater availability of habitats and food resources for forest bird species [82,83]. In our study area, the occurrence of *D*. *ochropyga* was strongly related with larger forest fragments and nuclear areas, which reinforces the idea that strictly forest birds respond negatively to the border effect, which is relatively lower in small fragments [84]. Some of the studied species forage in the soil, mainly litter, in search of insects [39,85]. Thus, changes caused by the edge effect can lead to a reduction in food availability as a result of alteration of the composition and density of the leaf layer [2]. Therefore, nuclear area size is one of the most effective indicators of the presence of forest species [63].

As expected, connectivity between fragments was strongly related to the occurrence of bird species in our study. The presences of five species (*D*. *ochropyga*, *F*. *serrana*, *M*. *leachii*, *S*. *scansor* and *X*. *fuscus*) were positively related to fragments with higher connectivity indexes. It is important to point out that the presence of a species with low dispersion capacity (*S*. *scansor*) [44] in highly-connected fragments corroborates our hypothesis. For species like *F*. *serrana* and *D*. *ochropyga* there have been no studies on the movement ability or on their ability to use open areas. However, our results indicated that these species presented the capacity for dispersion through forest patches low tolerance to open areas.

Finally, none of the tested variables of any of the three scales influenced the occurrence of two bird species, *M*. *leucoblephara* and *T*. *caerulescens*. Although both species are forest dependent, they possess low sensitivity to disturbance and are among the most generalists of the studied species [38,86], which may explain their lack of response.

Previous research has found that dispersal limitation is the dominant factor underlying the decline of insectivorous birds in fragmented tropical forests (e.g. [2,18,87–89]). Such studies support the hypothesis that insectivorous birds (particularly understory specialists) have low mobility and/or are reluctant to move through open habitats, potentially due to behavioral inhibition [44,88,90] or physiological/morphological limitations [2,91]. However, our findings are consistent with the hypothesis that dispersal limitation can be counterbalanced by the presence of a heterogeneous matrix with small fragments that function as stepping stones, thereby fostering population flow throughout the landscape [86,88,90], as was found for *X*. *fuscus* and *P*. *leucoptera*. Thus, isolation has been found not to have an influence on occupancy dynamics of many tropical birds [92], suggesting that habitat connectivity may not be the limiting factor in determining population dynamics in fragmented landscapes.

When the studied species are ranked according to their sensitivities to human disturbances (as observed in the study area, and described in the literature), we can speculate which species are more susceptible to local extinction due to forest fragmentation. We found two groups to be particularly threatened: those species that are very affected by forest fragmentation (*P*. *leucoptera*, *S*. *scansor* and *X*. *fuscus*), and those previously confirmed to occur in the region in other studies but were poorly observed during our surveys, potentially because their low density (*A*. *leucophthalmus*, *D*. *ochropyga* and *F*. *serrana*).

For the species *A*. *leucophthalmus*, *M*. *leachii*, *S*. *scansor* and *X*. *fuscus*, tree height influenced more their occurrence than vegetation density, therefore, to conserve these species it is important to conserve large trees in forests fragments. For *F*. *serrana*, the best predictors were vegetation density and connectivity, thus, the maintenance of the oldest fragments (the oldest forest fragments have a greater probability of having a well-developed understory) and the increase in the connectivity of the fragments by the formation of vegetation corridors would benefit the existence of this species. *P*. *leucoptera* needs bigger and more forested fragments to occur, therefore, a landscape with more forested areas would allow the occurrence of such species. For *D*. *ochropyga* the nuclear area proved to be more important as a predictive characteristic, indicating the possibility of a great impact on the edge effect for this species, thus requiring larger continuous protected areas. The specie *C*. *caudata* was associated with the large of the trees, indicating that this species is possibly less sensitive to landscape metrics, but associated with older forests.

## Conclusion

We investigated how habitat characteristics of multiple scales explain the occurrence of 10 different bird species among forest patches across a landscape in the Brazilian Atlantic Forest. The results found mixed effects at multiple scales and no single-scale model could effectively predict the occurrence of all ten species.

Tree height, one of the microhabitat scale characteristics, was the best in predicting the occurrence of five species (*A*. *leucophthalmus*, *D*. *ochropyga*, *M*. *leachii*, *S*. *scansor* and *X*. *fuscus*), whereas the local scale characteristic proportion of forest was the best predictor for the occurrence of two other species (*F*. *serrana* and *P*. *leucoptera*). In contrast, landscape characteristics were also significant, with AREA, CORE and dIIC influencing the occurrences of six species (*S*. *scansor*, *X*. *fuscus*, *D*. *ochropyga*, *P*. *leucoptera*, *F*. *serrana* and *M*. *leachii)*.

From a community perspective, the individual variables of the studied scales may be important for predicting the occurrence of some understory bird species, although the general response pattern of the studied understory bird community does not support a unique fragmentation threshold, the amount of habitat fragmentation supported by a given bird species. For a given variable, we observed both positive and negative relationships with species occurrence, highlighting an idiosyncratic response pattern across species. Because of these idiosyncratic responses among species, it would be difficult to implement a single, comprehensive management plan that addresses the specific habitat needs of each and every species. Instead, a focus on multiscalar management (or mosaic management) may provide more comprehensive guidance to land managers [93], allowing the evaluation of the contribution of fragments outside protected areas in the regional landscape [33]. The lack of a consensus in the species responses both within and among spatial scales may challenge simplistic and blueprint approaches [94].

Measuring landscape variables at more than one scale can also help ensure that the potential importance of landscape factors to species occurrence will not be missed, especially given that our analysis revealed that some species exhibited a relationship with characteristics of only one out of the three scales we assessed in this study. Although the natural vegetation of the Atlantic Forest is being altered at local and landscape scales, the wholesale environmental transformation of this region represents the greatest threat to most species at this time, and thus demands a multiscale approach to land management and species conservation. In this sense, a multiscale approach is necessary. This could involve a mosaic of different degrees of management and zoning protection, such as private reserves, city parks and squares, and strict conservation units, which would necessarily include different forests types of varying phytosociological structure. Additionally, biodiversity-friendly management of the landscape matrix is also important, including increasing overall forest-cover through the conservation of non-intensive systems, such as home-gardens, agroforestry operations and orchards. Finally, ecological models, as well as conservation practices, that rely on simplistic and single-scale frameworks are likely to make limited contributions to understanding and managing such a complex and nuanced world.

## Supporting information

S1 Table Sampling areas used to evaluate the occurrence of forest bird species in Ouro Preto municipality. Minas Gerais State. and southeastern Brazil. UTM coordinates (Zone 23).
(DOCX)

S2 Table Description of the variables used to evaluate microhabitat, local and landscape characteristics of the Atlantic Forest patches studied at Ouro Preto municipality. (DOCX)
