# Supplementary material for: Does scale matter? The influence of three-level spatial scales on forest bird occurrence in a tropical landscape
Source: PLoS One. 2018 Jun 18;13(6):e0198732. doi: 10.1371/journal.pone.0198732 (PMC6005493; doi:10.1371/journal.pone.0198732)
Supplement: S1 Table — Minas Gerais State. and southeastern Brazil. UTM coordinates (Zone 23). (DOCX) [file pone.0198732.s001.docx]

| Sampling sites | Area (ha) | Altitude (m) | Coordinates |  |
| --- | --- | --- | --- | --- |
| 1. Estação Ecológica do Tripuí (EET) * | 295.30 | 1233 | 652038.00 m E / 7745500.00 m S |  |
| 2. Parque Estadual do Itacolomi (PEIT) * | 5995.52 | 1348 | 655844.80 m E / 7740589.13 m S |  |
| 3. Parque Natural Municipal das Andorinhas (Andorinhas I) * | 18.17 | 1419 | 656270.00 m E / 7747330.00 m S |  |
| 4. Parque Natural Municipal das Andorinhas (Andorinhas II) * | 12.12 | 1364 | 656578.00 m E / 7746577.00 m S |  |
| 5. Parque Natural Municipal Horto dos Contos (Horto dos Contos) * | 5.58 | 1137 | 655901.00 m E / 7745450.00 m S |  |
| 6. Fazenda da Brígida (Brígida) | 139.04 | 1363 | 655372.00 m E / 7747552.00 m S |  |
| 7. Cadeia | 31.48 | 1178 | 653636.00 m E / 7745597.00 m S |  |
| 8. Campo | 12.12 | 1376 | 655785.27 m E / 7746457.38 m S |  |
| 9. Padre Faria | 13.32 | 1058 | 658202.00 m E / 7744862.00 m S |  |
| 10. Passa Dez | 10.09 | 1156 | 654052.00 m E / 7746209.00 m S |  |
| 11. Sede Campestre | 4.27 | 1062 | 656807.00 m E / 7743918.00 m S |  |
| 12. Trevo | 11.93 | 1242 | 652812.00 m E / 7746029.00 m S |  |
| 13. Vermelhão | 11.02 | 1184 | 653007.00 m E / 7742820.00 m S |  |
| *Protected Areas. | | | | |

**S1 Table.** Sampling areas used to evaluate the occurrence of forest bird species in Ouro Preto Municipality, Minas Gerais State, and southeastern Brazil. UTM coordinates (Zone 23)
