# Supplementary material for: Does scale matter? The influence of three-level spatial scales on forest bird occurrence in a tropical landscape
Source: PLoS One. 2018 Jun 18;13(6):e0198732. doi: 10.1371/journal.pone.0198732 (PMC6005493; doi:10.1371/journal.pone.0198732)
Supplement: S2 Table — (DOCX) [file pone.0198732.s002.docx]

**S2 Table.** Description of the variables used to evaluate microhabitat, local and landscape characteristics of the Atlantic Forest patches studied at Ouro Preto Municipality

| Scale | | Description |
| --- | --- | --- |
| Microhabitat | |  |
|  | TREE HEIGHT | Average tree heights. |
|  | DBH | Diameter at Breadth High |
|  | CANOPY COVER | Proportion of Canopy Cover |
| Local | |  |
|  | FOREST COVER (%) | Proportion of Forest Cover in a Buffer of 300 meters |
|  | URBANIZATION (%) | Proportion of Urbanization in a Buffer of 300 meters |
|  | RENDVI |  |
| Landscape | |  |
|  | AREA | Area of the fragment (ha) |
|  | CORE | Core area of the fragment with a 100 m edge |
|  | PROX | Index of proximity in a radius of 2000 m |
|  | ENN | Neighborhood distance – edge to edge . |
|  | dIIC | Delta of the integral index of connectivity |
